# 2-(5,6-Dihydro­benzimidazo[1,2-*c*]quinazolin-6-yl)-6-eth­oxy­phenol

**DOI:** 10.1107/S1600536811034714

**Published:** 2011-08-31

**Authors:** Naser Eltaher Eltayeb, Siang Guan Teoh, Kong Mun Lo

**Affiliations:** a School of Chemical Sciences, Universiti Sains Malaysia, Minden, Penang, Malaysia; b Department of Chemistry, International University of Africa, Sudan; c Chemistry Department, Faculty of Science, University of Malaya, Malaysia

## Abstract

In the title compound, C_22_H_19_N_3_O_2_, the phenol ring forms dihedral angles of 88.93 (10) and 87.95 (12)° with the benzimidazole system and the quinazoline benzene ring, respectively. In the crystal, mol­ecules are linked *via* O—H⋯N hydrogen bonds into infinite chains along [100]. An intra­molecular N—H⋯O hydrogen bond generates an *S*(6) ring.

## Related literature

For a related structure, references to our previous structural studies of similar compounds and background references to benzimidazoles, see: Eltayeb *et al.* (2011 ▶).
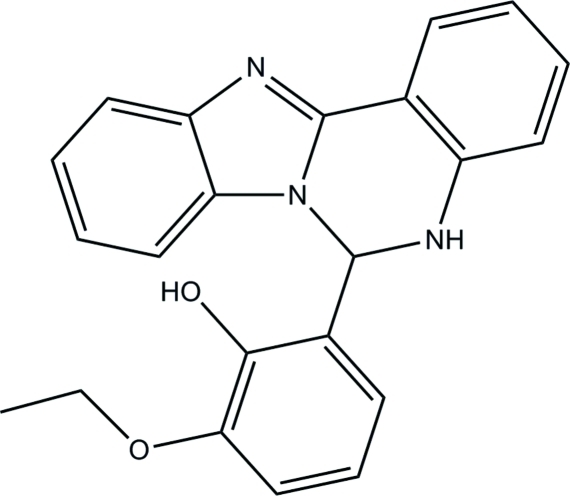


## Experimental

### 

#### Crystal data

C_22_H_19_N_3_O_2_
*M* *_r_* = 357.40
Triclinic, 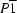

*a* = 8.6935 (3) Å
*b* = 10.9167 (3) Å
*c* = 11.3401 (5) Å
α = 107.193 (2)°
β = 108.923 (2)°
γ = 104.723 (2)°
*V* = 896.66 (6) Å^3^
*Z* = 2
Mo *K*α radiation
μ = 0.09 mm^−1^
*T* = 296 K
0.25 × 0.2 × 0.17 mm

#### Data collection

Bruker SMART APEXII CCD diffractometer
Absorption correction: multi-scan (*SADABS*; Bruker, 2009 ▶) *T*_min_ = 0.614, *T*_max_ = 0.746
7367 measured reflections
3505 independent reflections
2804 reflections with *I* > 2σ(*I*)
*R*_int_ = 0.022

#### Refinement

*R*[*F* ^2^ > 2σ(*F* ^2^)] = 0.059
*wR*(*F* ^2^) = 0.164
*S* = 1.00
3505 reflections
250 parameters
H atoms treated by a mixture of independent and constrained refinement
Δρ_max_ = 0.73 e Å^−3^
Δρ_min_ = −0.39 e Å^−3^

### 

Data collection: *APEX2* (Bruker, 2009 ▶); cell refinement: *SAINT* (Bruker, 2009 ▶); data reduction: *SAINT*; program(s) used to solve structure: *SHELXTL* (Sheldrick, 2008 ▶); program(s) used to refine structure: *SHELXTL*; molecular graphics: *SHELXTL*; software used to prepare material for publication: *SHELXTL* and *PLATON* (Spek, 2009 ▶).

## Supplementary Material

Crystal structure: contains datablock(s) I, global. DOI: 10.1107/S1600536811034714/hb6367sup1.cif

Structure factors: contains datablock(s) I. DOI: 10.1107/S1600536811034714/hb6367Isup2.hkl

Supplementary material file. DOI: 10.1107/S1600536811034714/hb6367Isup3.cml

Additional supplementary materials:  crystallographic information; 3D view; checkCIF report

## Figures and Tables

**Table 1:** Hydrogen-bond geometry (Å, °)

*D*—H⋯*A*	*D*—H	H⋯*A*	*D*⋯*A*	*D*—H⋯*A*
O1—H1*O*1⋯N1^i^	0.82	1.85	2.646 (3)	165
N2—H1*N*2⋯O1	1.01 (4)	2.12 (3)	2.811 (3)	124 (2)

Symmetry code: (i) 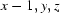.

**Fig. 1.**
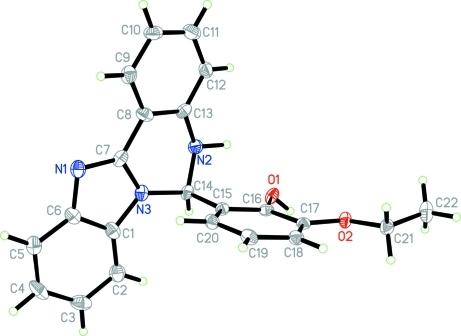
The molecular structure of (I) with 50% probability displacement ellipsoids.

**Fig. 2.**
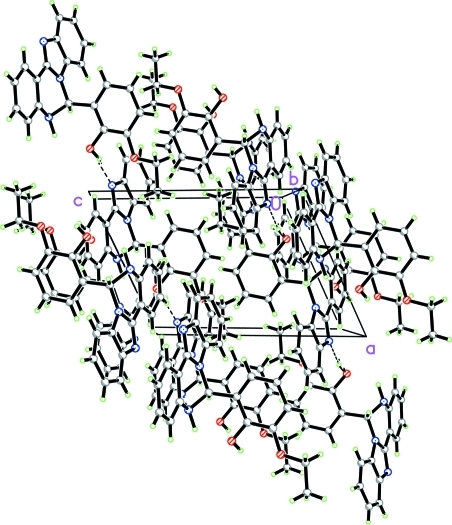
The crystal packing of the title compound viewed down the b axis. Hydrogen bonds are shown as dashed lines.

## supplementary crystallographic information

### Comment

As part of our ongoing structural studies of benzimidazoles and their derivatives (Eltayeb *et al.*, 2011, and references therein) we now describe in this paper the structure of the title compound, (I), (Fig. 1).

In the title compound, C_22_H_19_N_3_O_2_, the phenol-substituted ring forms dihedral angles of 88.93 (10) and 87.95 (12)° with the benzimidazole system and the quinazoline benzene ring, respectively. The phenol-substituted ring is perpendicular to the 10-membered quinazoline ring with dihedral angle 89.49 (9) °. The dihedral angle between the 9-membered benzimidazole and 10-membered quinazoline rings is 9.72 (8) °. The exthoxy group is planarly attached to the phenol ring.

In the crystal, molecules are linked *via* intermolecular O—H···N hydrogen bonds into infinite one-dimensional chain along [100]. The intramolecular N—H···O hydrogen bond generates an S(6) ring (Table 1).

C—H···π interactions are also present; C11—H11··· *Cg*5^i^ =2.63 Å, C20—H20···*Cg*1^ii^ = 2.79 Å and C21—H21A···*Cg*5^iii^ = 2.88 Å. *Cg*5 and *Cg*1 are centroids of C15—C20 and N1/C6/C1/N3/C7 rings respectively, [symmetry codes: (i) = *1-X,-Y,-Z*; (ii) = *X,Y,Z*; (iii) =*1-X,1-Y,1-Z*].

### Experimental

The title compound was synthesized by adding 3-ethoxthysalicylaldehyde (0.166 g, 1.0 mmol) to a solution of 2-(2-aminophenyl)-1*H*-benzimidazole 0.209 g, 1.0 mmol) in ethanol (30 ml). The color of the resulting solution is pale-yellow. Then upon adding zinc chloride (0.136 g, 1.0 mmol), the color of the solution became golden-yellow. The mixture was refluxed with stirring for 3 hrs. The resultant solution was filtered and the filtrate was evaporated to give a yellow solid product. Yellow blocks of (I) were obtained from ethanol by slow evaporation at room temperature after three weeks.

### Refinement

N bound H atom was located in a difference Fourier map and were refined freely. The remaining H atoms were positioned geometrically and refined using a riding model with C–H = 0.93 or 0.97 Å and *U*_iso_(H) = 1.2*U*_eq_(C) and *U*_iso_(H) = 1.5*U*_eq_(O). The highest residual electron density peak is 1.17Å from H2 and the deepest hole is 0.71Å from C1.

### Crystal data

**Table:**

C_22_H_19_N_3_O_2_	*Z* = 2
*M**_r_* = 357.40	*F*(000) = 376
Triclinic, *P*1	*D*_x_ = 1.324 Mg m^−^^3^
Hall symbol: -P 1	Mo *K*α radiation, λ = 0.71073 Å
*a* = 8.6935 (3) Å	Cell parameters from 2997 reflections
*b* = 10.9167 (3) Å	θ = 2.6–28.3°
*c* = 11.3401 (5) Å	µ = 0.09 mm^−^^1^
α = 107.193 (2)°	*T* = 296 K
β = 108.923 (2)°	Block, yellow
γ = 104.723 (2)°	0.25 × 0.2 × 0.17 mm
*V* = 896.66 (6) Å^3^	

### Data collection

**Table:**

Bruker SMART APEXII CCD diffractometer	3505 independent reflections
Radiation source: fine-focus sealed tube	2804 reflections with *I* > 2σ(*I*)
graphite	*R*_int_ = 0.022
φ and ω scans	θ_max_ = 26.0°, θ_min_ = 2.1°
Absorption correction: multi-scan (*SADABS*; Bruker, 2009)	*h* = −10→10
*T*_min_ = 0.614, *T*_max_ = 0.746	*k* = −13→13
7367 measured reflections	*l* = −13→13

### Refinement

**Table:**

Refinement on *F*^2^	Primary atom site location: structure-invariant direct methods
Least-squares matrix: full	Secondary atom site location: difference Fourier map
*R*[*F*^2^ > 2σ(*F*^2^)] = 0.059	Hydrogen site location: inferred from neighbouring sites
*wR*(*F*^2^) = 0.164	H atoms treated by a mixture of independent and constrained refinement
*S* = 1.00	*w* = 1/[σ^2^(*F*_o_^2^) + (0.0842*P*)^2^ + 1.0545*P*] where *P* = (*F*_o_^2^ + 2*F*_c_^2^)/3
3505 reflections	(Δ/σ)_max_ < 0.001
250 parameters	Δρ_max_ = 0.73 e Å^−^^3^
0 restraints	Δρ_min_ = −0.39 e Å^−^^3^

### Special details

**Table:**

Geometry. All e.s.d.'s (except the e.s.d. in the dihedral angle between two l.s. planes) are estimated using the full covariance matrix. The cell e.s.d.'s are taken into account individually in the estimation of e.s.d.'s in distances, angles and torsion angles; correlations between e.s.d.'s in cell parameters are only used when they are defined by crystal symmetry. An approximate (isotropic) treatment of cell e.s.d.'s is used for estimating e.s.d.'s involving l.s. planes.
Refinement. Refinement of *F*^2^ against ALL reflections. The weighted *R*-factor *wR* and goodness of fit *S* are based on *F*^2^, conventional *R*-factors *R* are based on *F*, with *F* set to zero for negative *F*^2^. The threshold expression of *F*^2^ > σ(*F*^2^) is used only for calculating *R*-factors(gt) *etc*. and is not relevant to the choice of reflections for refinement. *R*-factors based on *F*^2^ are statistically about twice as large as those based on *F*, and *R*- factors based on ALL data will be even larger.

### Fractional atomic coordinates and isotropic or equivalent isotropic displacement parameters (Å^2^)

**Table:**

	*x*	*y*	*z*	*U*_iso_*/*U*_eq_	
O2	0.2462 (2)	0.30314 (16)	0.30490 (16)	0.0186 (4)	
O1	0.3194 (2)	0.36858 (17)	0.10937 (16)	0.0198 (4)	
H1O1	0.2499	0.3974	0.1312	0.030*	
N2	0.5360 (3)	0.3168 (2)	−0.0210 (2)	0.0212 (4)	
N3	0.8203 (3)	0.4820 (2)	0.1277 (2)	0.0199 (4)	
C22	0.0082 (3)	0.1800 (3)	0.3379 (3)	0.0300 (6)	
H22A	−0.0427	0.2463	0.3258	0.045*	
H22B	−0.0237	0.1461	0.3992	0.045*	
H22C	−0.0350	0.1037	0.2510	0.045*	
C21	0.2056 (3)	0.2487 (2)	0.3970 (2)	0.0207 (5)	
H21A	0.2514	0.3232	0.4869	0.025*	
H21B	0.2584	0.1816	0.4059	0.025*	
C17	0.4186 (3)	0.3471 (2)	0.3259 (2)	0.0159 (5)	
C16	0.4501 (3)	0.3791 (2)	0.2227 (2)	0.0156 (5)	
C15	0.6199 (3)	0.4154 (2)	0.2305 (2)	0.0158 (5)	
C14	0.6409 (3)	0.4416 (2)	0.1107 (2)	0.0177 (5)	
H14	0.6012	0.5163	0.1025	0.021*	
C13	0.6001 (3)	0.2101 (2)	−0.0438 (2)	0.0196 (5)	
C8	0.7844 (3)	0.2458 (2)	0.0104 (2)	0.0210 (5)	
C9	0.8501 (3)	0.1412 (3)	−0.0197 (3)	0.0235 (5)	
H9	0.9712	0.1637	0.0156	0.028*	
C10	0.7343 (3)	0.0051 (2)	−0.1018 (3)	0.0264 (6)	
H10	0.7770	−0.0647	−0.1212	0.032*	
C18	0.5574 (3)	0.3556 (2)	0.4365 (2)	0.0181 (5)	
H18	0.5371	0.3364	0.5060	0.022*	
C19	0.7260 (3)	0.3924 (2)	0.4435 (2)	0.0193 (5)	
H19	0.8183	0.3978	0.5177	0.023*	
C20	0.7578 (3)	0.4213 (2)	0.3406 (2)	0.0184 (5)	
H20	0.8707	0.4444	0.3449	0.022*	
C11	0.5555 (3)	−0.0273 (3)	−0.1549 (3)	0.0276 (6)	
H11	0.4786	−0.1194	−0.2106	0.033*	
C12	0.4880 (3)	0.0721 (2)	−0.1280 (2)	0.0243 (5)	
H12	0.3665	0.0474	−0.1661	0.029*	
C7	0.8974 (3)	0.3931 (2)	0.0916 (2)	0.0193 (5)	
N1	1.0679 (3)	0.4543 (2)	0.1332 (2)	0.0214 (4)	
C6	1.1063 (3)	0.5952 (2)	0.2058 (2)	0.0202 (5)	
C5	1.2674 (3)	0.7073 (3)	0.2714 (2)	0.0228 (5)	
H5	1.3691	0.6958	0.2695	0.027*	
C4	1.2708 (3)	0.8375 (3)	0.3399 (3)	0.0312 (6)	
H4	1.3767	0.9147	0.3855	0.037*	
C3	1.1143 (4)	0.8535 (3)	0.3410 (3)	0.0303 (6)	
H3	1.1197	0.9411	0.3890	0.036*	
C2	0.9543 (3)	0.7420 (3)	0.2726 (3)	0.0257 (5)	
H2	0.8510	0.7523	0.2717	0.031*	
C1	0.9549 (3)	0.6149 (2)	0.2056 (2)	0.0222 (5)	
H1N2	0.409 (4)	0.280 (3)	−0.035 (3)	0.043 (9)*	

### Atomic displacement parameters (Å^2^)

**Table:**

	*U*^11^	*U*^22^	*U*^33^	*U*^12^	*U*^13^	*U*^23^
O2	0.0157 (8)	0.0213 (8)	0.0235 (8)	0.0083 (6)	0.0111 (7)	0.0116 (7)
O1	0.0160 (8)	0.0301 (9)	0.0206 (8)	0.0148 (7)	0.0098 (7)	0.0129 (7)
N2	0.0169 (10)	0.0223 (10)	0.0212 (10)	0.0076 (8)	0.0073 (8)	0.0062 (8)
N3	0.0174 (10)	0.0228 (10)	0.0222 (10)	0.0087 (8)	0.0097 (8)	0.0110 (8)
C22	0.0255 (13)	0.0367 (15)	0.0420 (15)	0.0142 (11)	0.0213 (12)	0.0250 (12)
C21	0.0246 (12)	0.0199 (11)	0.0237 (12)	0.0095 (9)	0.0141 (10)	0.0117 (9)
C17	0.0167 (11)	0.0114 (10)	0.0186 (11)	0.0066 (8)	0.0074 (9)	0.0040 (8)
C16	0.0178 (11)	0.0137 (10)	0.0162 (11)	0.0091 (8)	0.0067 (9)	0.0056 (8)
C15	0.0173 (11)	0.0134 (10)	0.0181 (11)	0.0076 (8)	0.0079 (9)	0.0065 (8)
C14	0.0164 (11)	0.0216 (11)	0.0226 (11)	0.0121 (9)	0.0107 (9)	0.0122 (9)
C13	0.0242 (12)	0.0247 (12)	0.0168 (11)	0.0144 (10)	0.0113 (10)	0.0106 (9)
C8	0.0279 (13)	0.0161 (11)	0.0140 (10)	0.0047 (9)	0.0066 (10)	0.0061 (9)
C9	0.0192 (12)	0.0267 (12)	0.0294 (13)	0.0120 (10)	0.0109 (10)	0.0147 (10)
C10	0.0370 (15)	0.0177 (12)	0.0274 (13)	0.0126 (10)	0.0168 (11)	0.0081 (10)
C18	0.0211 (11)	0.0158 (10)	0.0169 (11)	0.0057 (9)	0.0081 (9)	0.0072 (9)
C19	0.0161 (11)	0.0187 (11)	0.0167 (11)	0.0052 (9)	0.0014 (9)	0.0066 (9)
C20	0.0134 (11)	0.0182 (11)	0.0211 (11)	0.0061 (9)	0.0059 (9)	0.0066 (9)
C11	0.0325 (14)	0.0195 (12)	0.0263 (13)	0.0055 (10)	0.0127 (11)	0.0073 (10)
C12	0.0203 (12)	0.0244 (12)	0.0220 (12)	0.0026 (10)	0.0094 (10)	0.0063 (10)
C7	0.0198 (11)	0.0271 (12)	0.0178 (11)	0.0139 (10)	0.0095 (9)	0.0125 (10)
N1	0.0182 (10)	0.0255 (10)	0.0226 (10)	0.0091 (8)	0.0087 (8)	0.0125 (8)
C6	0.0217 (12)	0.0210 (11)	0.0152 (11)	0.0044 (9)	0.0048 (9)	0.0107 (9)
C5	0.0185 (12)	0.0310 (13)	0.0195 (11)	0.0085 (10)	0.0064 (9)	0.0142 (10)
C4	0.0291 (14)	0.0238 (13)	0.0233 (12)	−0.0064 (10)	0.0030 (11)	0.0107 (10)
C3	0.0426 (16)	0.0178 (12)	0.0272 (13)	0.0100 (11)	0.0131 (12)	0.0085 (10)
C2	0.0251 (13)	0.0269 (13)	0.0275 (13)	0.0097 (10)	0.0119 (11)	0.0138 (11)
C1	0.0215 (12)	0.0228 (12)	0.0202 (11)	0.0040 (9)	0.0068 (10)	0.0126 (10)

### Geometric parameters (Å, °)

**Table:**

O2—C17	1.367 (3)	C8—C7	1.461 (3)
O2—C21	1.438 (3)	C9—C10	1.379 (3)
O1—C16	1.362 (3)	C9—H9	0.9300
O1—H1O1	0.8200	C10—C11	1.375 (4)
N2—C13	1.407 (3)	C10—H10	0.9300
N2—C14	1.483 (3)	C18—C19	1.386 (3)
N2—H1N2	1.02 (3)	C18—H18	0.9300
N3—C7	1.354 (3)	C19—C20	1.386 (3)
N3—C1	1.401 (3)	C19—H19	0.9300
N3—C14	1.439 (3)	C20—H20	0.9300
C22—C21	1.503 (3)	C11—C12	1.364 (4)
C22—H22A	0.9600	C11—H11	0.9300
C22—H22B	0.9600	C12—H12	0.9300
C22—H22C	0.9600	C7—N1	1.312 (3)
C21—H21A	0.9700	N1—C6	1.403 (3)
C21—H21B	0.9700	C6—C1	1.385 (3)
C17—C18	1.389 (3)	C6—C5	1.389 (3)
C17—C16	1.402 (3)	C5—C4	1.391 (4)
C16—C15	1.394 (3)	C5—H5	0.9300
C15—C20	1.394 (3)	C4—C3	1.419 (4)
C15—C14	1.524 (3)	C4—H4	0.9300
C14—H14	0.9800	C3—C2	1.378 (4)
C13—C12	1.390 (3)	C3—H3	0.9300
C13—C8	1.414 (3)	C2—C1	1.373 (4)
C8—C9	1.404 (3)	C2—H2	0.9300
			
C17—O2—C21	116.69 (17)	C10—C9—H9	120.2
C16—O1—H1O1	109.5	C8—C9—H9	120.2
C13—N2—C14	117.46 (18)	C11—C10—C9	119.9 (2)
C13—N2—H1N2	111.6 (18)	C11—C10—H10	120.0
C14—N2—H1N2	109.0 (18)	C9—C10—H10	120.0
C7—N3—C1	106.87 (19)	C19—C18—C17	120.2 (2)
C7—N3—C14	125.18 (19)	C19—C18—H18	119.9
C1—N3—C14	126.96 (19)	C17—C18—H18	119.9
C21—C22—H22A	109.5	C20—C19—C18	120.4 (2)
C21—C22—H22B	109.5	C20—C19—H19	119.8
H22A—C22—H22B	109.5	C18—C19—H19	119.8
C21—C22—H22C	109.5	C19—C20—C15	119.7 (2)
H22A—C22—H22C	109.5	C19—C20—H20	120.1
H22B—C22—H22C	109.5	C15—C20—H20	120.1
O2—C21—C22	107.31 (19)	C12—C11—C10	121.7 (2)
O2—C21—H21A	110.3	C12—C11—H11	119.2
C22—C21—H21A	110.3	C10—C11—H11	119.2
O2—C21—H21B	110.3	C11—C12—C13	120.2 (2)
C22—C21—H21B	110.3	C11—C12—H12	119.9
H21A—C21—H21B	108.5	C13—C12—H12	119.9
O2—C17—C18	124.7 (2)	N1—C7—N3	113.7 (2)
O2—C17—C16	115.40 (19)	N1—C7—C8	128.5 (2)
C18—C17—C16	119.8 (2)	N3—C7—C8	117.8 (2)
O1—C16—C15	117.52 (19)	C7—N1—C6	104.17 (19)
O1—C16—C17	122.89 (19)	C1—C6—C5	120.5 (2)
C15—C16—C17	119.5 (2)	C1—C6—N1	110.7 (2)
C20—C15—C16	120.3 (2)	C5—C6—N1	128.8 (2)
C20—C15—C14	123.6 (2)	C6—C5—C4	117.4 (2)
C16—C15—C14	116.07 (19)	C6—C5—H5	121.3
N3—C14—N2	105.89 (17)	C4—C5—H5	121.3
N3—C14—C15	112.84 (18)	C5—C4—C3	120.6 (2)
N2—C14—C15	112.62 (18)	C5—C4—H4	119.7
N3—C14—H14	108.4	C3—C4—H4	119.7
N2—C14—H14	108.4	C2—C3—C4	121.4 (2)
C15—C14—H14	108.4	C2—C3—H3	119.3
C12—C13—N2	121.8 (2)	C4—C3—H3	119.3
C12—C13—C8	118.9 (2)	C1—C2—C3	116.7 (2)
N2—C13—C8	119.0 (2)	C1—C2—H2	121.7
C9—C8—C13	119.6 (2)	C3—C2—H2	121.7
C9—C8—C7	123.3 (2)	C2—C1—C6	123.3 (2)
C13—C8—C7	117.1 (2)	C2—C1—N3	132.2 (2)
C10—C9—C8	119.7 (2)	C6—C1—N3	104.5 (2)
			
C17—O2—C21—C22	−168.25 (19)	C16—C15—C20—C19	−0.8 (3)
C21—O2—C17—C18	−6.7 (3)	C14—C15—C20—C19	−178.3 (2)
C21—O2—C17—C16	170.40 (18)	C9—C10—C11—C12	−0.4 (4)
O2—C17—C16—O1	0.5 (3)	C10—C11—C12—C13	−0.7 (4)
C18—C17—C16—O1	177.77 (19)	N2—C13—C12—C11	175.7 (2)
O2—C17—C16—C15	−175.55 (18)	C8—C13—C12—C11	1.5 (4)
C18—C17—C16—C15	1.7 (3)	C1—N3—C7—N1	−2.4 (3)
O1—C16—C15—C20	−176.86 (19)	C14—N3—C7—N1	−171.7 (2)
C17—C16—C15—C20	−0.6 (3)	C1—N3—C7—C8	179.14 (19)
O1—C16—C15—C14	0.8 (3)	C14—N3—C7—C8	9.9 (3)
C17—C16—C15—C14	177.08 (18)	C9—C8—C7—N1	9.6 (4)
C7—N3—C14—N2	−37.3 (3)	C13—C8—C7—N1	−167.8 (2)
C1—N3—C14—N2	155.6 (2)	C9—C8—C7—N3	−172.1 (2)
C7—N3—C14—C15	86.4 (3)	C13—C8—C7—N3	10.4 (3)
C1—N3—C14—C15	−80.8 (3)	N3—C7—N1—C6	1.5 (3)
C13—N2—C14—N3	48.0 (3)	C8—C7—N1—C6	179.8 (2)
C13—N2—C14—C15	−75.8 (2)	C7—N1—C6—C1	0.0 (3)
C20—C15—C14—N3	−3.9 (3)	C7—N1—C6—C5	−179.5 (2)
C16—C15—C14—N3	178.50 (18)	C1—C6—C5—C4	2.3 (3)
C20—C15—C14—N2	115.9 (2)	N1—C6—C5—C4	−178.3 (2)
C16—C15—C14—N2	−61.7 (2)	C6—C5—C4—C3	−0.5 (4)
C14—N2—C13—C12	152.2 (2)	C5—C4—C3—C2	−1.2 (4)
C14—N2—C13—C8	−33.6 (3)	C4—C3—C2—C1	1.1 (4)
C12—C13—C8—C9	−1.2 (3)	C3—C2—C1—C6	0.8 (4)
N2—C13—C8—C9	−175.5 (2)	C3—C2—C1—N3	179.8 (2)
C12—C13—C8—C7	176.4 (2)	C5—C6—C1—C2	−2.5 (4)
N2—C13—C8—C7	2.0 (3)	N1—C6—C1—C2	177.9 (2)
C13—C8—C9—C10	0.1 (4)	C5—C6—C1—N3	178.2 (2)
C7—C8—C9—C10	−177.3 (2)	N1—C6—C1—N3	−1.4 (2)
C8—C9—C10—C11	0.7 (4)	C7—N3—C1—C2	−177.0 (3)
O2—C17—C18—C19	175.5 (2)	C14—N3—C1—C2	−8.0 (4)
C16—C17—C18—C19	−1.5 (3)	C7—N3—C1—C6	2.2 (2)
C17—C18—C19—C20	0.1 (3)	C14—N3—C1—C6	171.2 (2)
C18—C19—C20—C15	1.1 (3)		

### Hydrogen-bond geometry (Å, °)

**Table:**

*D*—H···*A*	*D*—H	H···*A*	*D*···*A*	*D*—H···*A*
O1—H1O1···N1^i^	0.82	1.85	2.646 (3)	165
N2—H1N2···O1	1.01 (4)	2.12 (3)	2.811 (3)	124 (2)

Symmetry codes: (i) *x*−1, *y*, *z*.
